# Collection and Evaluation of Genetic Diversity and Population Structure of Potato Landraces and Varieties in China

**DOI:** 10.3389/fpls.2019.00139

**Published:** 2019-02-21

**Authors:** Ying Wang, Muhammad Abdul Rehman Rashid, Xianping Li, Chunguang Yao, Lili Lu, Jianming Bai, Yanshan Li, Ningsheng Xu, Qiongfen Yang, Linhai Zhang, Glenn J. Bryan, Qijun Sui, Zhechao Pan

**Affiliations:** ^1^Industrial Crops Research Institute, Yunnan Academy of Agricultural Sciences, Kunming, China; ^2^Plant Breeding and Genetics, University of Agriculture Faisalabad, Burewala, Pakistan; ^3^Environment and Plant Protection Institute, Chinese Academy of Tropical Agricultural Sciences, Haikou, China; ^4^The James Hutton Institute, Invergowrie, United Kingdom; ^5^Scientific Observing and Experimental Station of Potato and Rapeseed in Yunnan-Guizhou Plateau, Ministry of Agriculture, Kunming, China

**Keywords:** potato, landraces, genetic diversity, population structure, SSR, domestication

## Abstract

China is the world’s leading country for potato production but potato is not native to China. To gain insights into the genetic diversity of potato germplasm various studies have been performed but no study has been reported for potato landraces in China. To improve the available genepool for future potato breeding programs, a diverse population containing 292 genotypes (including foreign elite lines, local landraces and cultivars) was developed and genotyped using 30 SSR markers covering the entire potato genome. A total of 174 alleles were detected with an average of 5.5 alleles per locus. The model-based structure analysis discriminated the population into two main sub-groups, which can be further subdivided into seven groups based on collection sites. One sub-group (P1) revealed less genetic diversity than other (P2) and contained a higher number of commercial cultivars possibly indicating a slight reduction in diversity due to selection in breeding programs. The P2 sub-group showed a wider range of genetic diversity with more new and unique alleles attained from wild relatives. The potato landraces, clustered in sub-population P1 may be derived from historical population imported from ancient European and International Potato Center genotypes while sub-population P2 may be derived from modern populations from International Potato Center and European genotypes. It is proposed that in the first step, the potato genotypes were introduced from Europe to China, domesticated as landraces, and then hybridized for modern cultivars.

## Introduction

The cultivated potato (*Solanum tuberosum* L.) was domesticated 8,000–10,000 years ago from diploid wild species (2*x* = 2*n* = 24) native to the Andes of Southern Peru (Spooner et al., 2005b). Its migration from the Andes to coastal Chile caused the adaptation to the long-day conditions and this improved potato germplasm later contributed greatly to the development of commercial cultivars worldwide (Hardigan et al., 2017). Since its domestication, it has been widely adopted into the human diet and has become the most important non-cereal staple food across the globe. Potato is an important food crop, serving as a major source of calories and food security in Asia and South America (Scott and Suarez, 2012). To feed the constantly increasing world population, it is important to improve the genetic potential of potato germplasm.

Being the world’s leading potato producer, China produced about 25% of the world’s potato production with 95.5 million tons in 2014 which increased to 99.1 million tons in 2016^1^. In China, potato breeding is mainly by conventional methods of selection and hybridization based on visual traits (Duan et al., 2009). Such morphological characters are normally vulnerable to environmental conditions and can lead to spurious improvement and slow progress. In China, the predominant potato cultivars were developed by manipulation of European tetraploid genotypes from 1950 to 1960 (Jian et al., 2017). Among the 288 cultivars released in China during 1950–2007, 34.7% (100), 32.3% (93), 18.4% (53) cultivars were derived from American, German and Polish genotypes, respectively, the remainder arose from CIP and Dutch cultivars (Duan et al., 2018). The use of a limited number of parental genotypes may have resulted in the narrow genetic base of present Chinese potato cultivars. Therefore, there is a need to find more diverse breeding material in China to broaden the genetic background of improved cultivars.

Around the mid-16th century, potato was first introduced to China but the exact entry route is still unclear. Historically, two possible routes have been proposed, the first being the introduction of potato to Beijing, Tianjin and Northern China by sea and then moved to the South and Southwestern part of China. The second proposed route is from Southeast Asia to Taiwan and then the coastal provinces of China as Fujian and Guangdong (Tong and Zhao, 1991). Alternatively, the second route may be from Southeast Asia to Myanmar and then enter to China from Yunnan province. Both of the routes may results in introduction of potato in Yunnan (the Southwestern province of China), which is one of the earliest provinces in China to grow potatoes. According to “Illustrated Catalog of Plants” written by Wu Qi-jun (1789–1847 AD) and published in 1848, Yunnan province had already planted different varieties (Sun et al., 2004). The ecological and climatic conditions of Yunnan province are similar to those of the center of potato origin, the Andes in South America, Peru and Chile. Old varieties introduced by missionaries into Yunnan and landrace diversification are well preserved as part of smallholder farming systems, in common with the Andes. Nevertheless, there has been no research to evaluate the genetic diversity among potato landraces in China and their contribution to improved cultivars. These landraces are still very popular because of their wide adaptability, unique flavor and good taste even with low yield. Usually these landraces are highly resistant to biotic stresses such as virus and late blight as smallholder farmers do not have access to virus free seeds. Therefore, it is very important to identify the genetic variation along with the genetic background of local and foreign genotypes and to develop the appropriate breeding program for improved potato cultivars to broaden the genetic background. Potato is naturally a cross-pollinated crop, which can be improved by exchange of favorable alleles between landraces and cultivars through hybridization. Therefore, the evaluation of genetic relationship among foreign elite lines, local landraces and improved cultivars is essential for successful exploitation, genetic stability and enhanced heterotic effect in the available germplasm. In a recent study, Duan et al. (2018) studied the genetic diversity among the alien and domestically improved cultivars but present study is the first to report the genetic variability and the contribution of locally adapted potato landraces in Chinese cultivars.

Among DNA markers, simple sequence repeats (SSR) have been used successfully in polyploid species such as *Brassica napus* (Hasan et al., 2008; Xiao et al., 2012), Arachis (Huang et al., 2012), sweet potato (Yang et al., 2015) and potato species (Ghislain et al., 2009; Duan et al., 2018). SSR markers have been preferred due to their random genome distribution, high level of polymorphism, simplicity of use, high clarity and reproducibility, low operational cost, hyper-variability, amenability to automation, ease of multiplexing and use with low quality DNA (Ghislain et al., 2009; Yang et al., 2015; Jian et al., 2017; Duan et al., 2018). SSR markers have been widely used in determination of genetic diversity, germplasm fingerprinting, heterosis analysis, tracing germplasm migrations, gene flow, genetic linkage mapping and phylogenetic studies.

This study reports on the genetic diversity of potato cultivars and their progenitors; foreign elite lines and local landraces in China. We collected a diverse germplasm of 292 potato (*S. tuberosum* L.) genotypes, an appropriate panel for potato breeding programs in China. The genetic structure and relationship among landraces and cultivars was evaluated to select the suitable parental lines for genetic improvement by increasing heterotic effects and base-broadening. We also presented a model of potato dispersal and enhancement as a possible route of introduction and evolution of European genotypes in China.

## Materials and Methods

### Plant Materials

A collection of 292 potato genotypes from International Potato Center (CIP), Europe and different agro-ecological regions of China was collected. The selected germplasm comprised 137 foreign and 155 domestic genotypes. Among the 137 foreign genotypes, 87 genotypes were from CIP and 50 genotypes were from Europe (America, Belarus, Germany, and Netherlands), while 155 local genotypes included 65 landraces from Yunnan province of China, 30 improved varieties from Northern China, 25 from Southwestern China and 35 local commercial cultivars from YAAS (Yunnan Academy of Agricultural Sciences). The material from CIP belongs to modern LTVR (Low Tropic Virus Resistant) and classical B3 populations of CIP known as CIP-C and CIP-D at China, respectively. The detailed information of the geographical distribution of 292 genotypes listed in Supplementary Table S1.

### SSR Genotyping

At least three individual plants of each genotype were selected, and a bulk of young leaves was harvested to obtain high quality DNA. Whole genomic DNA was extracted using Qiagen DNeasy kit according to the manufacturer’s protocol. The template DNA concentration was quantified by nanodrop2000c and diluted to 10 ng per μL for further analysis. A set of 30 SSR primer pairs with 2–3 primer per chromosome coverage (Table 1) with stable and clear amplifications as reported previously (Milbourne et al., 1998; Ghislain et al., 2004, 2009; Feingold et al., 2005) were used to genotype the 292 potato genotypes. SSR amplification was performed using PCR in a 25 μL reaction volume, containing 10 ng genomic DNA, 12.5 μL 2 × Taq PCR Master Mix (TIANGEN, China), 1 μL of each primer (10 μM), and 8.5 μL ddH_2_O. Thermal cycling conditions were 94°C for 4 min, 33 cycles of 94°C for 1 min, primer specific annealing temperature (Tm) for 1min, and 72°C for 1 min, followed by a final extension of 4 min at 72°C. The size base separation of PCR products was performed by the QIAxcel Advanced System (QIAGEN, Germany). The new Process Profile with OM1200 running method was used. The 10 s injection time and single run per row was followed with DNA High Resolution gel cartridge. The 15–600 bp QX alignment marker and 25 bp-500bp QX size marker were used for sample selection. The fragment size was recorded by the built-in software on the machine automatically. Each polymorphic fragment was scored as 1 or 0 for the presence or absence of amplification, respectively. Different SSR alleles were then named using the primer name and the fragment size.

**Table 1:** Description of 30 SSR markers used in this study to evaluate the genetic diversity in 292 potato genotypes.

Code	Name	Map location	Repeat motif	GenBank#, accession#	Primer sequences	T^o^a	Size (bp)
M1	^a^STM1049	I dg	(ATA)n	X13497	CTACCAgTTTgTTgATTgTggTg AgggACTTTAATTTgTTggACg	54 (57)	197–219
M2	^d^STG0016	I g	(AGA)n	BI178934	AgCTgCTCAgCATCAAgAgA ACCACCTCAggCACTTCATC	55 (53)	137–174
M3	^d^STM5127	I eg	(TCT)n	[M23e7]	TTCAAgAATAggCAAAACCA CTTTTTCTgACTgAgTTgCCTC	55 (60)	248–291
M4	^a^STM2022	II deg	(CAA)n…(CAA)n	[C112]	gCgTCAgCgATTTCAgTACTA TTCAgTCAACTCCTgTTgCg	58 (53)	173–243
M5	^a^STM1064	II deg	(TA)n (TG)n GT (TG)n	AC215425	gTTCTTTTggTggTTTTCCT TTATTTCTCTgTTgTTgCTg	55 (55)	201–213
M6	^d^STM5114	II eg	(ACC)n	[M102B19]	AATggCTCTCTCTgTATgCT gCTgTCCCAACTATCTTTgA	60 (57)	297–322
M7	^a^STM1053	III dg	(TA)n (ATC)n	AB022690	TCTCCCCATCTTAATgTTTC CAACACAgCATACAgATCATC	53 (53)	170–196
M8	^d^STG0010	III g	(TG)n	BM407152	CgATCTCTgCTTTgCAggTA gTTCATCACTACCgCCgACT	60 (55)	175–192
M9	^e^STM3023	IV	(GA)9 (GA)8 (GA)4		AAGCTGTTACTTGATTGCTGCA GTTCTGGCATTTCCATCTAGAGA	50	169–201
M10	^c^STI0001	IV fg	(AAT)n	CK860917	CAgCAAAATCAgAACCCgAT ggATCATCAAATTCACCgCT	60 (55)	194–215
M11	^c^STI0012	IV f	(ATT)n	U69633	gAAgCgACTTCCAAAATCAgA AAAgggAggAATAgAAACCAAAA	56 (55)	183–234
M12	^b^STMoAc58	V eg	(TA)n	X55749	TTgATgAAAggAATgCAgCTTgTg ACgTTAAAgAAgTgAgAgTACgAC	- (57)	243–263
M13	^c^STI0032	V fg	(GGA)n	BQ120452	TgggAAgAATCCTgAAATgg TgCTCTACCAATTAACggCA	61 (60)	127–148
M14	^a^STM0019	VI dg	AT)n (GT)n (AT)n (GT)n (GC)n (GT)n	[MAC33]	AATAggTgTACTgACTCTCAATg TTgAAgTAAAAgTCCTAgTATgTg	- (47)	99–206
M15	^c^STI0004	VI fg	(AAG)n	BQ118939	GCTgCTAAACACTCAAgCAgAA CAACTACAAgATTCCATCCACAg	60 (55)	83–126
M16	^a^STM0031	VII dg	(AC)n…(AC)n GCAC (AC)n (GCAC)n	[MAC50]	CATACgCACgCACgTACAC TTCAACCTATCATTTTgTgAgTCg	53 (57)	185–211
M17	^e^STM2013	VII	(TCTA)6		TTCGGAATTACCCTCTGCC AAAAAAAGAACGCGCACG	55	146–172
M18	^c^STI0033	VII fg	(AGG)n	BG886969	TgAgggTTTTCAgAAAgggA CATCCTTgCAACAACCTCCT	61 (60)	131–155
M19	^a^STM1104	VIII deg	(TCT)n	EU548082	TgATTCTCTTgCCTACTgTAATCg CAAAgTggTgTgAAgCTgTgA	53 (57)	178–199
M20	^c^STI0003	VIII fg	(ACC)n	AW096896	ACCATCCACCATgTCAATgC CTCATggATggTgTCATTgg	60 (55)	137–188
M21	^a^STM3012	IX d	(CT)n (CT)n	[61D9]	CAACTCAAACCAgAAggCAAA gAgAAATgggCACAAAAAACA	56 (57)	180–225
M22	^a^STM1052	IX eg	(AT)n GT (AT)n (GT)n	AJ133765	CAATTTCgTTTTTTCATgTgACAC ATggCgTAATTTgATTTAATACgTAA	50 (52)	214–263
M23	^c^STI0014	IX fg	(TGG)n (AGG)n	BQ115461	AgAAACTgAgTTgTgTTTgggA TCAACAgTCTCAgAAAACCCTCT	54 (55)	127–157
M24	^a^STM1106	X dg	(ATT)n	X95821	TCCAgCTgATTggTTAggTTg ATgCgAATCTACTCgTCATgg	51 (55)	145–211
M25	^d^STG0025	X g	(AAAC)n	BQ506618	TggAATCCgAATTACgCTCT AggTTTTACCACTCgggCTT	56 (55)	208–223
M26	^d^STG0001	XI g	(CT)n	BE340539	CAgCCAACATTTgTACCCCT ACCCCCACTTgCCATATTTT	58 (52)	137–163
M27	^a^STM0037	XI dg	(TC)n (AC)n AA (AC)n (AT)n	[MAC62]	AATTTAACTTAgAAgATTAgTCTC ATTTggTTgggTATgATA	52 (53)	87–133
M28	^a^STM0030	XII deg	comMound(GT/GC) (GT)n	[MAC05]	AgAgATCgATgTAAAACACgT gTggCATTTTgATggATT	58 (53)	122–168
M29	^c^STI0030	XII fg	(ATT)n	BF188393	TTgACCCTCCAACTATAgATTCTTC TgACAACTTTAAAgCATATgTCAgC	58 (60)	94–137
M30	^d^STM5121	XII g	(TGT)n	[M46L17]	CACCggAATAAgCggATCT TCTTCCCTTCCATTTgTCA	48 (50)	297–309


*The sources of markers were indicated with superscript on top left of marker names as ^*a*^for Milbourne et al. (1998); ^*b*^for Ghislain et al. (2004); ^*c*^for Feingold et al. (2005); ^*d*^for Ghislain et al. (2009), and ^*e*^for Ghislain et al. (2006)*.

### Data Analysis

The software Popgene version 1.32^2^ was used to estimate the *Na* (observed number of alleles per locus), *Ne* (effective number of alleles per locus), *H* (Nei’s genetic diversity), *I* (Shannon’s information index), number of polymorphic alleles, total number of alleles, and percentage of polymorphic alleles (%). The genetic diversity (GD) and polymorphic information content (PIC) were estimated by PowerMarker v3.25 (Liu and Muse, 2005) to evaluate the discriminatory power of different primers.

The model-based program Structure v2.4.2 (Pritchard et al., 2000) was used to analyze the population structure of the 292 potato genotypes by using 174 alleles of 30 SSR primer pairs. Ten independent simulations were carried out for each *K* (the number of populations) ranging from 1 to 10. For each simulation, 10,000 iterations before a burn-in length of 50,000 MCMC (Markov Chain Monte Carlo) replications were performed with the selection of admixture and related frequency models. The LnP(D) values and optimal *K-*value was estimated using Evanno’s Δ*K* method (Evanno et al., 2005) with online tool Structure Harvester (Earl, 2012).

The neighbor joining (NJ) method based on Nei’s genetic distances among genotypes (Nei, 1978) using DARwin ver. 6 (Perrier and Jacquemoud-Collet, 2006) was followed for cluster analysis of germplasm. The tree was visualized and edited by Evloview online tool (He et al., 2016). The pairwise genetic distances among the Sub-groups were estimated by NTSYS-pc ver. 2.10e (Rohlf, 2000). The analysis of molecular variance (AMOVA) was performed by GeneAlEx-6.5 (Peakall and Smouse, 2006, 2012) to find the genetic differentiation among 292 potato genotypes.

## Results

### Marker Polymorphism

Thirty SSR markers distributed over all 12 potato chromosomes were used to genotype the entire population of 292 genotypes (Table 1). A total of 174 polymorphic alleles was detected. A high richness of alleles was observed with an average of 5.8 alleles per primer pair, ranging from 3 to 9 alleles per primer pair. The effective alleles per locus (Ne) ranged from 1.098 to 1.709 while Nei’s gene diversity (H) ranged from 0.087 to 0.400, and Shannon’s information index (I) ranged from 0.179 to 0.586 (Table 2).

**Table 2:** Genetic diversity parameters of 30 SSR markers evaluated in 292 potato genotypes as whole population and sub-populations.

Marker	NA^∗^	Ne^∗^	H^∗^	I^∗^	Polymorphic alleles	Total alleles	% Polymorphic alleles	Whole population		Subpopulation P1		Subpopulation P2	
								GD^∗^	PIC^∗^	GD	PIC	GD	PIC
M1	2	1.297	0.199	0.323	7	7	100	0.2834	0.2264	0.2973	0.2351	0.2485	0.2020
M2	2	1.469	0.295	0.449	6	6	100	0.3269	0.2547	0.3248	0.2547	0.3271	0.2538
M3	2	1.236	0.175	0.303	8	8	100	0.2765	0.2281	0.2974	0.2423	0.2356	0.2009
M4	2	1.334	0.223	0.363	9	9	100	0.3103	0.2517	0.2298	0.1792	0.2703	0.2204
M5	2	1.154	0.129	0.246	4	4	100	0.2250	0.1955	0.2155	0.1892	0.2316	0.1998
M6	2	1.496	0.275	0.408	4	4	100	0.2876	0.2302	0.2707	0.2197	0.2987	0.2365
M7	1.833	1.245	0.153	0.247	5	6	83	0.2012	0.1621	0.0886	0.0781	0.2363	0.1895
M8	2	1.259	0.134	0.200	4	4	100	0.1068	0.0908	0.1225	0.0995	0.0905	0.0801
M9	2	1.550	0.346	0.527	7	7	100	0.4543	0.3503	0.3143	0.2565	0.4818	0.3656
M10	2	1.564	0.344	0.521	4	4	100	0.4373	0.3396	0.4389	0.3404	0.4305	0.3361
M11	1.889	1.205	0.137	0.228	8	9	89	0.1931	0.1588	0.1904	0.1569	0.1935	0.1590
M12	2	1.098	0.087	0.179	4	4	100	0.1587	0.1427	0.1155	0.1062	0.1881	0.1652
M13	2	1.574	0.342	0.516	5	5	100	0.3793	0.3014	0.4126	0.3242	0.3422	0.2735
M14	2	1.271	0.204	0.351	8	8	100	0.3264	0.2679	0.2030	0.1667	0.3305	0.2661
M15	2	1.333	0.217	0.347	7	7	100	0.2471	0.1979	0.2681	0.2151	0.2280	0.1824
M16	2	1.465	0.301	0.472	4	4	100	0.4119	0.3246	0.3140	0.2509	0.4195	0.3285
M17	2	1.709	0.393	0.574	6	6	100	0.3847	0.3088	0.3090	0.2524	0.4167	0.3273
M18	2	1.311	0.193	0.307	5	5	100	0.2518	0.1995	0.2712	0.2181	0.2318	0.1813
M19	2	1.486	0.314	0.489	5	5	100	0.4298	0.3352	0.3755	0.2940	0.4330	0.3379
M20	2	1.684	0.396	0.583	6	6	100	0.4558	0.3506	0.4357	0.3398	0.4467	0.3460
M21	2	1.474	0.305	0.475	6	6	100	0.4133	0.3246	0.3333	0.2656	0.4149	0.3268
M22	2	1.405	0.273	0.438	7	7	100	0.3911	0.3114	0.2732	0.2255	0.4412	0.3431
M23	2	1.215	0.146	0.244	5	5	100	0.2112	0.1710	0.1884	0.1513	0.2267	0.1841
M24	2	1.479	0.288	0.443	7	7	100	0.3609	0.2851	0.2891	0.2312	0.3677	0.2910
M25	2	1.706	0.400	0.586	4	4	100	0.3468	0.2733	0.3181	0.2530	0.3646	0.2856
M26	2	1.562	0.333	0.505	6	6	100	0.3954	0.3143	0.3859	0.3060	0.3993	0.3177
M27	2	1.638	0.352	0.506	5	5	100	0.3796	0.2900	0.3221	0.2550	0.3346	0.2644
M28	2	1.495	0.319	0.494	7	7	100	0.4322	0.3371	0.4266	0.3343	0.4106	0.3231
M29	1.667	1.238	0.151	0.237	4	6	67	0.2026	0.1596	0.2018	0.1601	0.2020	0.1584
M30	2	1.270	0.208	0.358	3	3	100	0.3365	0.2763	0.3450	0.2829	0.3291	0.2702


*^∗^Na, observed Number of Alleles per Locus; ^∗^Ne, effective number of alleles per locus; ^∗^I, Shannon’s information index; ^∗^H, Nei’s gene diversity; ^∗^GD, genetic diversity; ^∗^PIC, polymorphic information contents.*

### Population Structure

**FIGURE 1:**
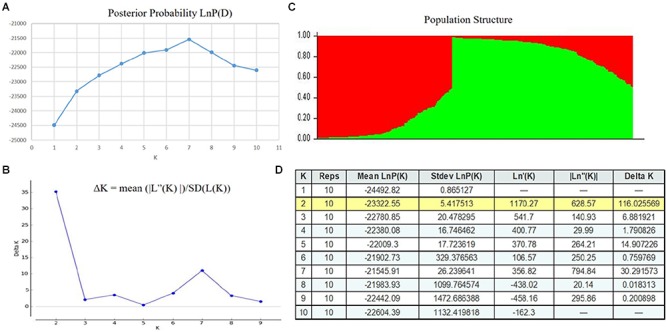
Population Structure of 292 diverse genotypes, the graphical presentation of estimation of posterior probability **(A)** and ΔK **(B)**, as well as tabulated values **(D)** and, *Q*-values based population structure **(C)** of 292 diverse potato genotypes with 1–10 K populations. The colored region grouped the genotypes in corresponding populations as Red (P1) and Green (P2).

The posterior probability of data, the LnP(D) scores for the number of populations (*K*) increased continuously from 1 to 7 and showed the inflation point at K7 which subdivides the whole panel into seven subgroups (Figure 1A). However, the Δ*K*-value rapidly decreased at *K* = 2 (Figure 1B–D), meanwhile, it showed the second peak at *K* = 7, indicating the whole population can be divided into two sub-populations which could be further subdivided into seven groups. It has been reported that if the model criterion continues to increase with increasing *K*-value, which capture most of the structure in the data which it seems biologically sensible (Pritchard et al., 2000). Therefore, we divided the 292 genotypes into two sub-populations, P1 with 126 and P2 with 166 genotypes. Both of the populations further subdivided into seven classes as their collection sites; Yunnan local cultivars, Chinese Southwestern cultivars, Chinese Northern cultivars, Chinese Landraces from Yunnan, European cultivars, modern/non-commercial CIP material (CIP-C), ancient/commercial CIP material (CIP-D). The 91% (115/126) and 93% (154/166) for genotypes in Sub-population P1 and P2 mainly contributed by five and four subgroups, respectively (Supplementary Table S2). Sub-population P1 contained 126 genotypes, of which 16 genotypes were local landraces, and 8 cultivated genotypes of Yunnan province of China, 28 from Northern and 18 from Southwestern China while 56 were from outside China, 37 from International Potato Center (CIP) and 19 from Europe or North America. In sub-population P2, out of total 166 genotypes 49 and 27 were local landraces and cultivated genotypes of Yunnan, 2 and 7 genotypes from northern and southwestern China while 47 and 31 genotypes were from CIP and Europe or North America, respectively.

### Genetic Diversity in 292 Potato Germplasm

The genetic diversity (GD) in the whole population ranged from 0.1068 to 0.4558 with an average of 0.309 while PIC ranged from 0.0908 to 0.3506 with an average of 0.2467. In sub-population P1, the GD and PIC reduced as from 0.0886 to 0.4389 with an average of 0.2784 and, 0.0781 to 0.3404 with an average of 0.2235, respectively. Nevertheless, the sub-population P2 showed the wider range of GD (0.0905–0.4818) and PIC (0.0801–0.3656) than the whole population with similar means as 0.306 and 0.2444, respectively (Supplementary Table S3).

### Phylogenetic Analysis

**FIGURE 2:**
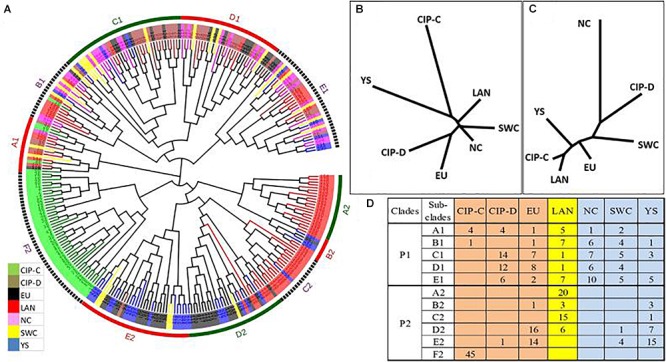
Structural characterization of 292 diverse genotypes. **(A)** Phylogenetic tree of all 292 genotypes estimated by 174 alleles of 30 SSR markers, the clades 1 and 2 represent the sub-populations P1 and P2, respectively, while sub-population P1 subdivided by alphabets A–E and sub-population P2 subdivided by alphabets A–F. **(B)** The dendrogram for various groups in P1 and **(C)** in P2. **(D)** The region based frequency of genotypes in various groups of Sub-populations. Hence, Eu, European genotypes; LAN, Landraces in China; NC, Varieties from Northern China; SWC, Varieties from Southwest of China; YS, local cultivars.

Akin to population structure analysis, the phylogenetic analysis also classified the whole panel into two sub-populations as P1 and P2 (Figure 2A). The seven subgroups were further mixed in both populations indicating the highly complex nature of potato domestication in China. For a better understanding of genetic evolution of seven sub-groups in populations, the phylogenetic relationship based on Nei’s distance for P1 and P2 was evaluated separately (Figure 2B,C).

In sub-population P1 (Figure 2B) maximum genetic distance (0.3602) was revealed by CIP-C and CIP-D genotypes. The Northern and Southwestern genotypes showed the closest relation with minimum genetic distance (0.076) and they were closely related to landraces (0.0896) (Table 3). The genetic distance of Southwestern genotypes from European genotypes was greater (0.1557) than their distance from CIP-D (0.1506) and landraces (0.119). Among the alien genotypes, the landraces showed a lesser distance from European genotypes (0.1541) than that of CIP-D (0.1714) and CIP-C (0.279) genotypes.

**Table 3:** Genetic distances among different groups in sub-population P1 (up diagonal), and sub-population P2 (down diagonal).

	CIP-C	CIP-D	^∗^EU	^∗^LAN	^∗^NC	^∗^SWC	^∗^YS
CIP-C	0	0.3602	0.3176	0.279	0.2431	0.2382	0.3586
CIP-D	0.3641	0	0.1479	0.1714	0.1212	0.1506	0.2182
EU	0.1157	0.363	0	0.1541	0.1129	0.1557	0.268
LAN	0.0494	0.3601	0.1152	0	0.0896	0.119	0.2014
NC	0.7674	0.9141	0.7561	0.7453	0	0.076	0.1859
SWC	0.218	0.547	0.22	0.1959	0.7368	0	0.2382
YS	0.1209	0.4082	0.1269	0.1313	0.8058	0.2546	0


*^∗^Eu, European genotypes; ^∗^LAN, landraces; ^∗^NC, genotypes form Northern China; ^∗^SWC, genotypes form Southwest of China; ^∗^YS, local cultivars*.

The range of genetic distances among genotypes in sub-population P2 was wider (0.0494 - 0.9141) than in P1 (0.076-0.3602). The closest relationship was observed between CIP-C and LAN while the maximum genetic distance was revealed by CIP-C and CIP-D genotypes. Unlike in P1, Northern genotypes showed wider genetic distance from Southwestern genotypes (0.7368) and local cultivars (0.8058). The cultivars exhibited the closest relationship to CIP-C (0.1209) followed by European genotypes (0.1269) and landraces (0.1313). The European genotypes revealed the nearby relationship to landraces while the landraces have almost similar distances for European genotypes and the local cultivars.

Both populations further grouped the genotypes into five and six clades named A1, B1, C1, D1, E1 and, A2, B2, C2, D2, E2 and F2 for P1 and P2, respectively (Figure 2A). In Sub-population P2 the clade A2 contained 20 landraces which were grouped closely with three landraces, one European genotype and three local cultivars in clade B2. In clade C2, 15 landraces may be have been exchanged the genetic material by hybridization with European genotypes of D2 and E2 to develop local genotypes of Northern and Southwestern China in D2 and E2. All these genotypes in clades A2 and E2 were rooted together with 45 CIP-C genotypes in F2 (Figure 2D).

### Population Differentiation Analysis

The genetic differentiation among population was revealed by analysis of molecular variances which indicated that the major proportion (90%, *P* < 0.001) of molecular variance was attributed to variation within population while 10% of the total molecular variance in germplasm were attributed to among populations (Supplementary Table S4).

## Discussion

### Genetic Diversity in Potato Germplasm

Previous efforts to explore the genetic diversity in Chinese potato germplasm as done by Duan et al. (2018), focused on local and foreign cultivars. The genetic diversity of Chinese potato landraces has not been reported previously. However, the genetic diversity in domesticated germplasm especially of potato has a key role for the proficient exploration of useful alleles existing in landraces and diverse genotypes. As a cross-pollinated species, potato has potential to exchange the favorable alleles among the landraces and improved cultivars. Conventional methods for characterizing potato germplasm based on phenotypic assessment of agronomic traits were laborious, time-consuming and could be influenced by environmental factors (Yang et al., 2015).

Molecular markers have been used to explore the population structure and genetic diversity of various crops. Peculiarly in potato, the marker-assisted selection (Ghislain et al., 1999), high-resolution mapping (Meksem et al., 1995), fingerprinting for intellectual property rights claims (McGregor et al., 2000; Duan et al., 2018) genetic diversity (Hoque et al., 2013; Siddappa et al., 2014; Onamu et al., 2016) and phylogenetic studies (Spooner et al., 2005a) were based on AFLP, RFLP, RAPD, and SSR markers. Among molecular marker types, in present research, SSRs were often selected for their high polymorphism level and reproducibility. Different researchers have used SSR markers for evaluating the genetic diversity in potato (Ispizúa et al., 2007; Fu et al., 2009; Kandemir et al., 2010; de Galarreta et al., 2011; Juyó et al., 2015). A decade ago, the “Potato Genetic Identity Kit” (PGI) of 24 SSRs based on fingerprinting of potato landraces was introduced for diversity evaluation (Ghislain et al., 2009). Total 30 SSR primer pairs, including 24 from the aforementioned PGI-kit and six others, were employed in this study. We observed 174 alleles with 3 to 9 alleles being detected per primer pair with an average of 5.8. This average of SSR alleles per primer was higher than detected in previous studies as 4.07 for 380 diverse genotypes of sweet potato (Yang et al., 2015), and 2.05 alleles per primer for sweet potato landraces and cultivars derived from polycross breeding (Hwang et al., 2002). We considered that amplified fragments from a primer pair arose from a single locus to suit the statistical analysis. It is prevalent method for polyploidy species such as *B. napus* (Hasan et al., 2008; Xiao et al., 2012), *Arachis* (Huang et al., 2012) and sweet potato (Yang et al., 2015). The wider range of gene diversity and PIC values in P2 sub-population may indicate the polycross derived genotypes originated from diverse genetic resources, which have not been done in P1 sub-population. This variation in genetic background also observed in alleles-richness among genotypes (Zhao et al., 2013).

The decrease in the number of diverse genotypes in sub-population P1 caused a reduction of genetic diversity. In sub-population P2 the wider range of GD and PIC than the whole population showed the availability of distant neighbor as CIP, Europe and China-origin-landraces in this population. Hence, the selection of parental lines from the P2 sub-population may induce the new alleles and enhance the heterotic effect of improved cultivars.

### Population Structure and Genetic Differentiation

In the previous studies, the cluster analysis only with the potato cultivars remained unable to differentiate the genetic back ground of the genotypes (Duan et al., 2018). However, the 292 genotypes were clustered into two sub-populations using structure analysis, and the results were consistent with results of phylogenetic evaluation. This may provide confirmation of differentiation and relationship among populations. The genetic differentiation can be evaluated by genetic diversity (Chen et al., 1997). Our results showed an apparent variation in PIC and genetic diversity score among populations (Table 2). For further confirmation, the highly significant (*P* < 0.001) genetic differentiation between populations was demonstrated by AMOVA (Supplementary Table S4).

The population structure divided the genotypes from seven collection sites into two main sub-populations, which still can be differentiated into seven groups by their collection sites. It may indicate the gene flow between the origins and collection sites. As the hybridization between genotypes and reintroduction of landraces also contributes to the genetic diversity (Camadro, 2012). The two sub-populations clearly indicated the preferred parental lines from various collection sites in historical breeding program, which continuously manipulated and caused a narrower genetic base of improved cultivars. However, the non-preferred lines were still involved in cultivar development indicating the availability of some favorable alleles, which can be explored by further studies.

The degree of genetic relationship and differentiation provide information about the different genetic background of potato genotypes. Therefore, the selection of genetically distant genotypes for hybridization in potato breeding programs will potentially lead to elite varieties with broadened genetic bases. These results indicated the great potential of accelerating the genetic improvement in the future potato-breeding programs by marker-based selection. The wider range of genetic diversity (Table 2) and genetic distances (Table 3) among the genotypes of P2 may indicate the potential gene pool for future potato breeding programs. It will not only lead to the genetic improvement of potato genotypes but also will use to explore the new alleles for valuable agronomic traits.

### Dispersal and Enhancement of Chinese Potato Landraces and Cultivars

Potato is not a native Chinese crop species but was introduced in the last half of the 18^th^ century (Xiang, 2018). The domestication-based classification of genotypes was also studied and a clear pattern was observed. Out of 292 genotypes, the 137 were from foreign sources and 90 were the local cultivars and improved varieties. Whereas, the majority of the landraces (49 of 65) belong to P2 and majority of local cultivars (54 of 90) belong to P1. Our analysis revealed that P1 landraces were favored and selected for local breeding programs to develop commercial cultivars and elite varieties.

The majority of landraces (49 of 65) showed a closed relationship with foreign genotypes (81 of 137) in P2 while 16 out of 65 landraces showed a close relationship to 56 foreign genotypes in P1. The P1 genotypes included 54 improved cultivars of this study but P2 contained only 36. Combined the results with pairwise differentiation among sub-populations and the groups within subpopulations (Table 3), it can be proposed that the majority of potato landraces of Yunnan province may have originated from Europe and migrated to Southern-China and with the passage of time evolved and enhanced as local landraces. The similar evolutionary model without any scientific evidence has been proposed previously (Tong and Zhao, 1991). It is known that, from 1934 to 1945, the 14 varieties, 62 hybrid seed combinations from Britain and the United States were introduced to China. From this material about six varieties, such as Shengli and Katadine etc., were selected and popularized (Stevenson, 1948). From 1950s to 1970s, most germplasm sources of foreign varieties were introduced from East Germany, Poland and the former Soviet Union. From 1980s to 1990s, more than 100 improved clones and 140 hybrid-combinations seeds were introduced from CIP (Lu and Xie, 2014). These germplasm were further crossed and spread to the broader regions of China, these were further selected, hybridized, and improved as modern cultivars for commercialization. Most of these landraces selected by human included in P1 and the majority of historical (28 Northern and 18 Southwestern) cultivars and a few (8 Yunnan) modern cultivars in P1 were derived from CIP-D and ancient/commercially known European genotypes (Supplementary Table S2).

After 1995, with the increase of international exchanges and the development of potato processing industry, potato was introduced from the Netherlands, the United States, Canada, Russia, Belarus, and other countries and CIP. In recent years, China has imported over 8000 accessions from CIP (Lu and Xie, 2014). Therefore, it was found that the majority (27 of 35) of modern cultivars were derived from CIP-C and modern/non-commercial European parental lines (Supplementary Table S2). It is also important to notice that the landraces, which were grouped in P2, due to any of the reasons, may be more closely related to the wild relatives. The landraces, which were not widely selected for commercial breeding may not have the preferred traits but may useful for mining the new alleles for the traits of agronomic importance and, biotic and abiotic resistance.

The potato originated from Andes in South America, Peru, and Chile adapted to short-day conditions. Later it was spread to European countries and adapted there to long-day conditions. In ancient history, this potato germplasm introduced from Europe to Northern part of China and further transferred to Southwestern China. It is akin to our finding as the P1 subpopulation contains maximum cultivars from Northern (28 cultivars) and Southwestern (18 cultivars) China derived from the ancient material from CIP (CIP-D) and Europe (Europe–America). The CIP and China chronicles the cooperation and exchange since 1980s (Lu and Xie, 2014). After 1980s, China directly can import the short-day adapted germplasm from CIP. That is observable in our findings that P2 subpopulation contained majority of modern Yunnan cultivars (27 cultivars) those may derived from the improved CIP (CIP-C) and modern European materials.

To confirm these evolutionary results and to infer the history of potato in China, the genetic distinction among various groups was studied. A credible evolutionary model was observed using 174 alleles amplified from 30 SSR markers in potato germplasm. If we classify the genotypes based on population structure, P1 and P2 have 4 and 7 unique alleles relative to each other, respectively, which transferred from European and CIP-D genotypes to P1 landraces and, from European and CIP-C genotypes to P2 landraces. The landraces showed the closest relationship to European and CIP-C genotypes in P1 and P2, respectively. The relationship of modern cultivars and elite varieties of Northern and Southwestern China indicated the possession of maximum alleles from domesticated landraces, which mainly were collected from Southwestern province (Yunnan) of China for this research. It endorsed the introduction of European and CIP-C genotypes to Yunnan and from here spread to the other areas of China.

The results of allele distribution and genetic distance among foreign genotypes, landraces and local cultivars supported the alleged hypothesis that modern elite varieties and commercial cultivars were originated from landraces, which were further selected and enhanced after the introduction of foreign genotypes to Southwestern China. These results were supported by the previous study reporting the introduction of the Irish potato, sweet potato, pineapple from America to China (Goodrich, 1938). This study provided the first molecular marker based scientific evidence to support the historical account of potato introduction in China. However, the limited number of molecular markers and biased sampling were inevitable in making biased inferences in this study. Further study may needed with a wider range of sampling with a greater number of markers to establish the clear footprint of potato evolution in China.

## Author Contributions

YW designed the experiments. MR analyzed data and wrote the manuscript. XL, CY, LL, JB, YL, NX, and QY helped in material collection and data analysis. LZ and GB reviewed and edited the manuscript. ZP and QS conceived and supervised the project.

## Conflict of Interest Statement

The authors declare that the research was conducted in the absence of any commercial or financial relationships that could be construed as a potential conflict of interest.

## Footnotes

1 http://faostat.fao.org/

2 https://sites.ualberta.ca/\tildefyeh/popgene.pdf
